# Black-White Inequities in Perception of Access to Neighborhood Resources Among Older Adults

**DOI:** 10.1093/geroni/igaa057.2472

**Published:** 2020-12-16

**Authors:** Michael Esposito, Dominique Sylvers, Philippa Clarke, Jessica Finlay, Joy Bohyun Jang, Sandra Tang

**Affiliations:** University of Michigan, Ann Arbor, Michigan, United States

## Abstract

Drawing on insights from critical race scholarship, this study examines how access to neighborhood resources varies among black and white older adults. Using Bayesian multilevel models, we estimate how evaluations of one’s neighborhood environment (e.g., perceived access to parks) varies by race, conditional on objective environmental measures (e.g., park area in one’s neighborhood). Results suggest that within the same spatial areas, individuals occupying marginalized statuses are less likely to perceive their neighborhoods as providing sufficient/accessible collective goods. Conditional on living in tracts with equal public park infrastructure, for instance, black respondents are 15% [95-CI: 8%, 22%] more likely to describe their neighborhoods as “lacking accessible parks.” Results suggest that these inequities are further exacerbated by race-related structural features (e.g., residential segregation) and other markers of welfare and marginalization (e.g., cognitive function). Overall, findings suggest that access to neighborhood-resources—and the benefits they confer—are fashioned by broader systems of power and inequality.

